# Assessing amblyopia treatment using multifocal visual evoked potentials

**DOI:** 10.1186/s12886-018-0877-0

**Published:** 2018-08-13

**Authors:** Junwon Jang, Sungeun E. Kyung

**Affiliations:** Department of ophthalmology, University of Dankook, Dankook University Hospital, 359 Manghang-Ro, Dongnam-Gu, Cheonan-City, Chungchungnam-Do South Korea

**Keywords:** mfVEP (multifocal visual evoked potential), Anisometropia, Amblyopia, Amplitude, Latency

## Abstract

**Background:**

To evaluate the effect of occlusion treatment for anisometropic amblyopia using multifocal visual evoked potentials (mfVEPs).

**Methods:**

The patients for this study comprised 19 patients (mean age 6.05 ± 1.65 years) with anisometropic amblyopia underwent mfVEP analysis using the RETIscan® system before and after occlusion treatment. After dividing the area into six ring areas and four quadrants, we analyzed the amplitudes and latencies of the mfVEPs.

**Results:**

The amplitudes of ring 1 (central field) in amblyopic eyes after treatment were significantly higher than those in the other rings (*p* = 0.001). The mfVEP amplitudes in each of the six rings between amblyopic eyes and fellow eyes at diagnosis and after occlusion treatment showed no significant differences. In quadrant 1 the amplitudes of the amblyopic eyes and fellow eyes were significantly different at the time of diagnosis (*p* = 0.005), whereas after occlusion treatment there was no significant difference (*p* = 0.888). The amplitudes for each of the six rings at diagnosis and after occlusion treatment in amblyopic eyes versus fellow eyes showed no significant difference. There were also no differences in the amplitudes in each of the four quadrants at the time of diagnosis and after occlusion treatment in amblyopic eyes versus fellow eyes. No significant difference was found in the comparison of latency values in each of the six rings or in each of the four quadrants at diagnosis and after occlusion treatment in amblyopic eyes versus their fellow eyes.

**Conclusions:**

The amplitudes of quadrant 1 in amblyopic eyes compared with those of the fellow eyes at diagnosis were increased after occlusion treatment. Changes of the difference between amblyopic eyes and fellow eyes in quadrant 1 after occlusion treatment could be a useful, objective method for monitoring improvement in visual acuity.

**Electronic supplementary material:**

The online version of this article (10.1186/s12886-018-0877-0) contains supplementary material, which is available to authorized users.

## Background

Amblyopia is a developmental loss of visual sensitivity caused by experiencing discordant binocular images early in life. It specifically refers to a decrease in best-corrected visual acuity in an eye with no organic pathology [1]. Amblyopia is commonly associated with visual deprivation, anisometropia, and strabismus. Anisometropic amblyopia is a decrease in the best-corrected visual acuity in one eye that results from considerably different refractive errors in the patient’s eyes. The eye that provides a more blurred image to the retina, and subsequently the brain, develops amblyopia [2].

Most anisometropic amblyopia patients in therapy are children due to the urgency of the critical development window, and occlusion (i.e. patching, atropinization) of the fellow eye is the usual tratment.^2^ Also, treatments including refractive correction, and atropine eye drops to the fellow eye have been shown to improve the vision of the amblyopic eye [3].

Because most of the patients are children, it has been necessary to develop objective methods in addition to measuring visual acuity to monitor vision after treatment with occlusion. Visual evoked potentials are commonly used for this purpose. Conventional visual evoked potentials testing in all types of amblyopia yields abnormal results [4, 5].

Relative to the normal eye, the decrease of visual acuity and contrast sensitivity in the amblyopic eye is far more significant in the fovea than at the periphery of the visual field [6, 7]. Standard visual evoked potentials do not provide topographic information in the retino-cortical pathway, limiting information about topographic differences in processing. This information might be overcome by employing a multifocal stimulation technique. Multifocal visual evoked potentials are used to investigate pathological or functional changes in the visual system, and specifically as a diagnostic tool for optic neuritis, glaucoma, amblyopia and ischemic optic neuropathy [8–11]. For recording multifocal visual evoked potentials, adjacent locations in the visual field are stimulated simultaneously with temporally uncorrelated stimuli. Individual responses in the visual field are extracted using cross-correlation methods [12].

In the present study, we evaluated the effect of occlusion treatment of unilateral anisometropic amblyopia using the multifocal visual evoked potentials technique. The purpose of our study is to validate the use of the multifocal visual evoked potentials in amblyopia management.

## Methods

### Patients

A retrospective chart review of patients diagnosed with unilateral anisometropic amblyopia, who performed multifocal visual evoked potentials at the university medical center from March 2013 to May 2015, was conducted. This study has been granted an exemption from Dankook university ethics approval and it was conducted according to the tenets of the Declaration of Helsinki. The patients for this study comprised 19 patients (mean age 6.05 ± 1.65 years) with anisometropic amblyopia who underwent multifocal visual evoked potentials testing at Dankook University at the time of diagnosis and again after occlusion treatment. The recording was usually carried out between 10 AM and 3 PM. Amblyopia was diagnosed on the basis of a clear history after the age of 4 to < 12 years. A difference of at least one diopter of anisometropia and a difference of two Snellen lines were required at diagnosis. None had either unsteady foveal or eccentric fixation. The best-corrected visual acuity was measured in each eye using Snellen chart or Tumbling E chart. Protocol-specified follow-up visits for occlusion were conducted 6 weeks after spectacle correction. Patients were prescribed two continuous hours of daily patching with spectacle correction until the difference in the best-corrected visual acuity between the eyes must be less than two lines. We followed them for average 12 months (6 months -18 months).

Inclusion criteria for unilateral anisometropic amblyopes were as follows: clear cornea and lens, no ocular pathology and no oculomotor disorders such as strabismus or nystagmus. The difference in the best-corrected visual acuity between the eyes must be less than two lines in the amblyopic eyes after occlusion treatment were included. Two patients with poor attendance (unexplained missing visits or no documented follow up) or poor compliance (failure of spectacle correction or 2 h occlusion) were excluded. Follow-up examination was performed after at least 6 months. Patient information is given in Table 1. Institutional review board approval was not required for this study.

**Table 1:** Demographic characteristics of the participants

Case	Sex	Refractive error(D)		BCVA^a^ (At diagnosis)		BCVA (After treatment)	
		Amblyopic eye	Fellow eye	Amblyopic eye	Fellow eye	Amblyopic eye	Fellow eye
1	M	sph + 5.5	sph + 0.75	20/30	20/20	20/20	20/20
2	M	sph + 2.5,cyl + 1.5	sph + 1.5	20/30	20/15	20/20	20/15
3	M	sph + 4.5, cvl + 2.0	sph + 3.5, cyl + 1.5	20/40	20/25	20/20	20/20
4	F	sph + 0.5, cyl − 3.5	sph + 0.25, cyl − 2.0	20/25	20/15	20/20	20/15
5	F	sph + 4.0	sph + 0.5	20/30	20/20	20/20	20/20
6	M	sph + 5.5	sph + 0.25	20/30	20/20	20/20	20/20
7	F	sph + 3.0, cyl −1.0	sph + 1.0	20/30	20/20	20/20	20/20
8	F	sph + 2.0, cyl-4.5	sph + 0.5	20/30	20/15	20/20	20/15
9	F	sph + 5.0, cyl + 1.0	sph + 3.0, cyl + 1.0	20/30	20/20	20/20	20/15
10	F	sph + 1.5, cyl −2.5	sph + 1.0	20/30	20/20	20/20	20/20
11	F	sph + 4.5, cyl-6.0	sph + 0.5	20/100	20/15	20/20	20/15
12	F	sph + 1.0, cyl + 1.5	sph + 1.25	20/40	20/20	20/20	20/20
13	F	sph + 6.0	sph + 2.0	20/30	20/20	20/20	20/20
14	F	sph −1.75	sph −0.5	20/30	20/20	20/20	20/20
15	M	sph + 4.5	sph + 1.0	20/40	20/15	20/20	20/15
16	M	sph + 3.75	sph + 2.75	20/70	20/20	20/20	20/20
17	F	sph + 3.0, cyl −4.0	sph + 2.25	20/30	20/15	20/20	20/15
18	M	sph + 2.0	sph + 0.25	20/50	20/20	20/20	20/20
19	M	sph + 7.0	sph + 5.0	20/50	20/20	20/20	20/20

^a^*BCVA* = Best corrected visual acuity

*D*: Diopter

### Multifocal visual evoked potential test procedure and analysis

The multifocal visual evoked potentials test was conducted using the RETIscan® system (Roland, Brandenburg, Germany). The distance to the 21-in. color cathode ray tube monitor was 30 cm, which corresponded to a total angular subtense of 60°. The stimulus was comprised of 60 checkerboard sections, most effective among all the check sizes, each containing eight white and eight black alternating squares [13]. Luminance of the white squares was 200 cd/m^2^, and that of the black squares was < 1 cd/m2, producing a Michelson contrast of 99%. Background luminance of the screen was maintained at the maximal level of 200 cd/m^2^. The visual stimuli were generated on a computer screen with a refresh rate of 50 Hz, and the pattern of reversals for each quadrant followed a pseudo-random sequence.

Gold cup electrodes were placed on the occipital scalp using electroencephalography paste to minimize impedance below 5KΩ. These electrode placements were based on the four-channel recording of Klistorner and Graham [14]. This modified, four-channel recording is currently the most widely used technique for recording multifocal visual evoked potentials. Two active electrodes were placed along the vertical midline 4 cm above the inion and 3 cm below the inion. Two more active electrodes were placed 4 cm on either side of the inion. A forehead electrode placed at the glabella served as the reference electrode and an earlobe electrode served as the ground electrode.

To measure the amplitude of responses, a signal to noise ratio (SNR) was calculated across the interval from 0 to 500 msec by specifying signal window (0 to 200 msec) and a “noise-only” window (300 to 500 msec). The amplification gain was ±100 μV. The low- and high-frequency cutoffs were set at 1 and 30 Hz, respectively. The signals were bandpass-filtered at 50 and 100 Hz, respectively. The same stimuli were administered to all subjects with the same electrode positions to obtain the multifocal visual evoked potentials responses. The extracted waveforms were analyzed via the best visual evoked potentials response method. The best visual evoked potentials response method is the custom-designed program of RETIscanⓇ which selecting the waveform of maximal amplitude among various waveforms recorded in each channel [15].

Before amblyopia treatment, the patients were tested with their manifest refractive correction. They were instructed to maintain their fixation on the center of the stimulus. The non-tested eye was patched. Two runs were completed for each eye in a right−left−right−left sequence, always beginning with the right eye. The averaged data from the two trials were analyzed. During recording, the position of the stimulated eye was monitored constantly by the examiner via the camera display provided in the RETIscan. Recording segments contaminated by fixation loss, unsteady fixation or external noises were discarded.

**Fig. 1:**
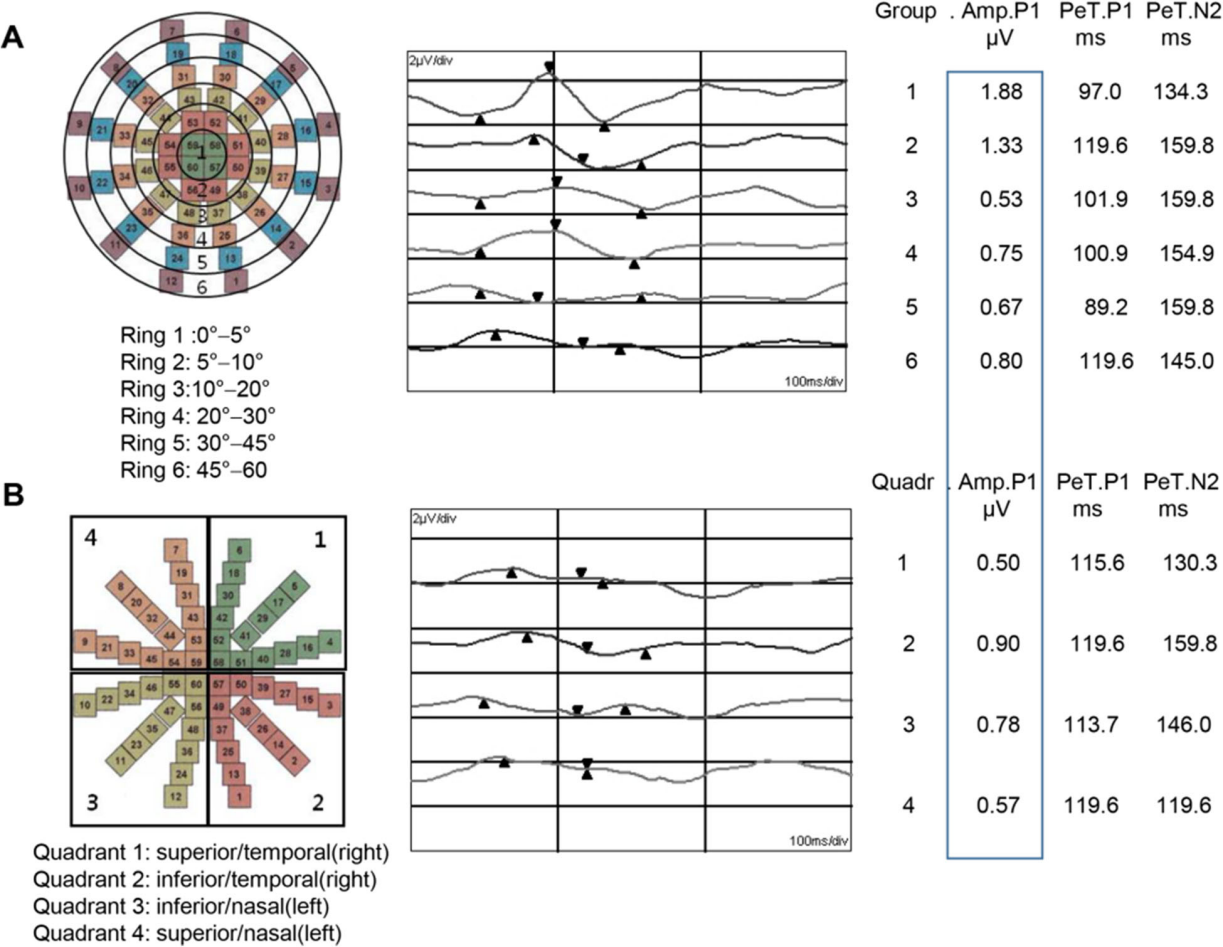
Diagrams and waveforms show the six rings (**a**) and four quadrants (**b**) on visual fields of multifocal visual evoked potentials (The box on the figure shows the amplitude values as an example)

In a field divided into 60 areas, we analyzed the mean amplitudes and the mean latencies of each topographical region from two trials, with six ring-shaped areas and four quadrants. The six rings were divided by their distance from the foveal center: ring 1, 0−5°; ring 2, 5°−10°; ring 3, 10°−20°; ring 4, 20°−30°; ring 5, 30°−45°; ring 6, 45°−60 (Fig. 1). The four quadrants were also divided according to the horizontal meridian and the vertical midline: quadrant 1, superior and temporal (right); quadrant 2, inferior and temporal (right); quadrant 3, inferior and nasal (left); quadrant 4, superior and nasal (left) (Fig. 1). After amblyopia treatment the multifocal visual evoked potentials testing was repeated (see additional file 1).

### Statistical analysis

All statistics were calculated using SPSS version 18.0 software (SPSS Inc., Chicago, IL, USA). The Friedman test was performed to identify any significant differences among the six rings and four quadrants in the amblyopic eyes at the time of diagnosis and after occlusion treatment. The Wilcoxon sign rank test was performed to identify any significant differences for each ring and each quadrant between the time of diagnosis and after occlusion treatment, as well as between the amblyopic and fellow eyes. A value of *p* < 0.05 was considered to indicate statistical significance.

## Results

The 19 patient cohort showed significant visual acuity changes from the pre-treatment baseline tests. The mean visual acuity change was from 0.27 ± 0.15 to 0 ± 0 in logMAR (Table 1).

### Amplitude

**Fig. 2:**
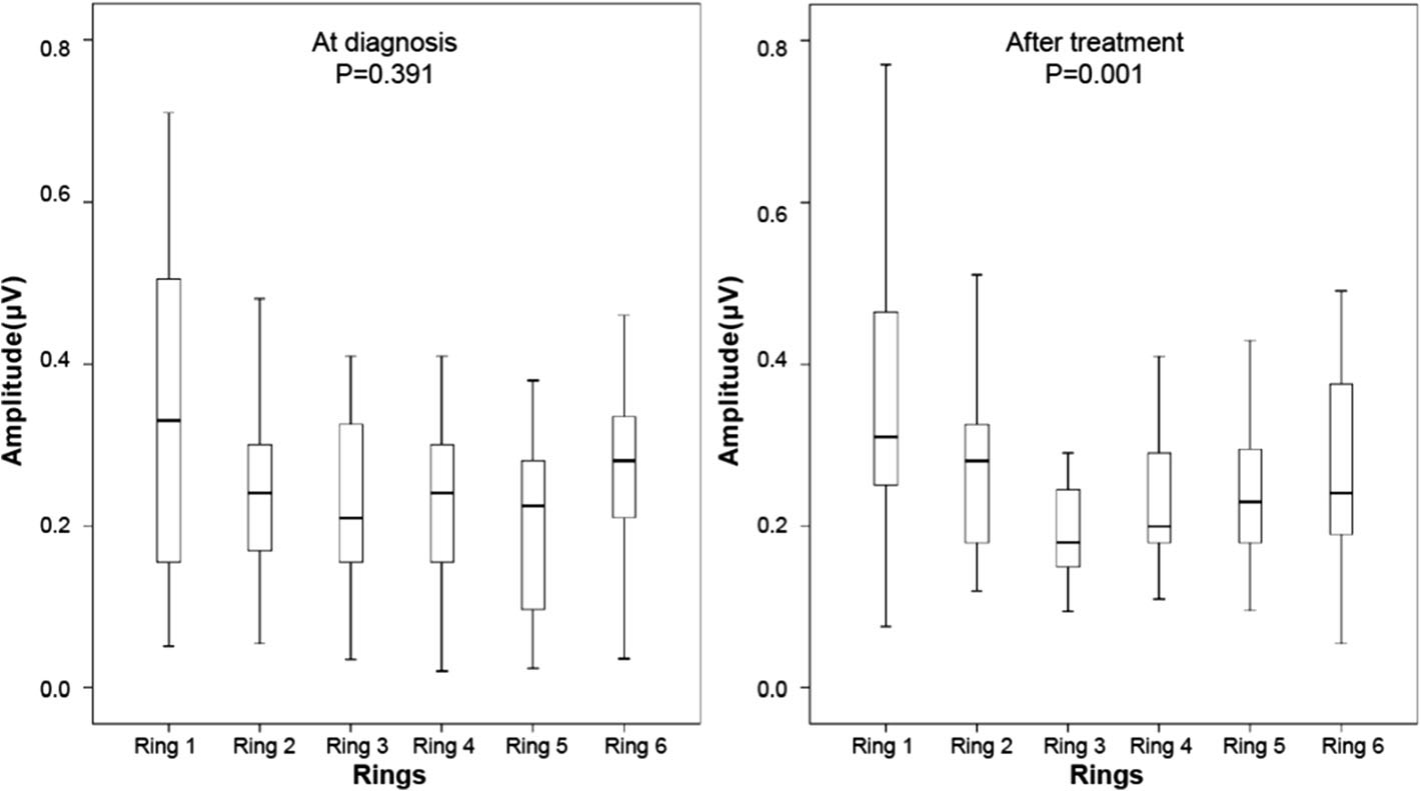
Box plots of data show the mean amplitude of multifocal visual evoked potentials in rings at diagnosis and after occlusion treatment of amblyopia. In amblyopic eyes, the values of the rings did not differ significantly at the time of diagnosis (*p* = 0.391, Friedman test). After treatment, however, the value in ring 1 (central field) was significantly higher (*p* = 0.001, Friedman test) than that of the other rings (Wilcoxon test): rings 1 vs. 2 (*p* = 0.042); rings 1 vs. 3 (*p* = 0.003); rings 1 vs. 4 (*p* = 0.006), rings 1 vs. 5 (*p* = 0.004); rings 1 vs. 6 (*p* = 6 0.033)

**Fig. 3:**
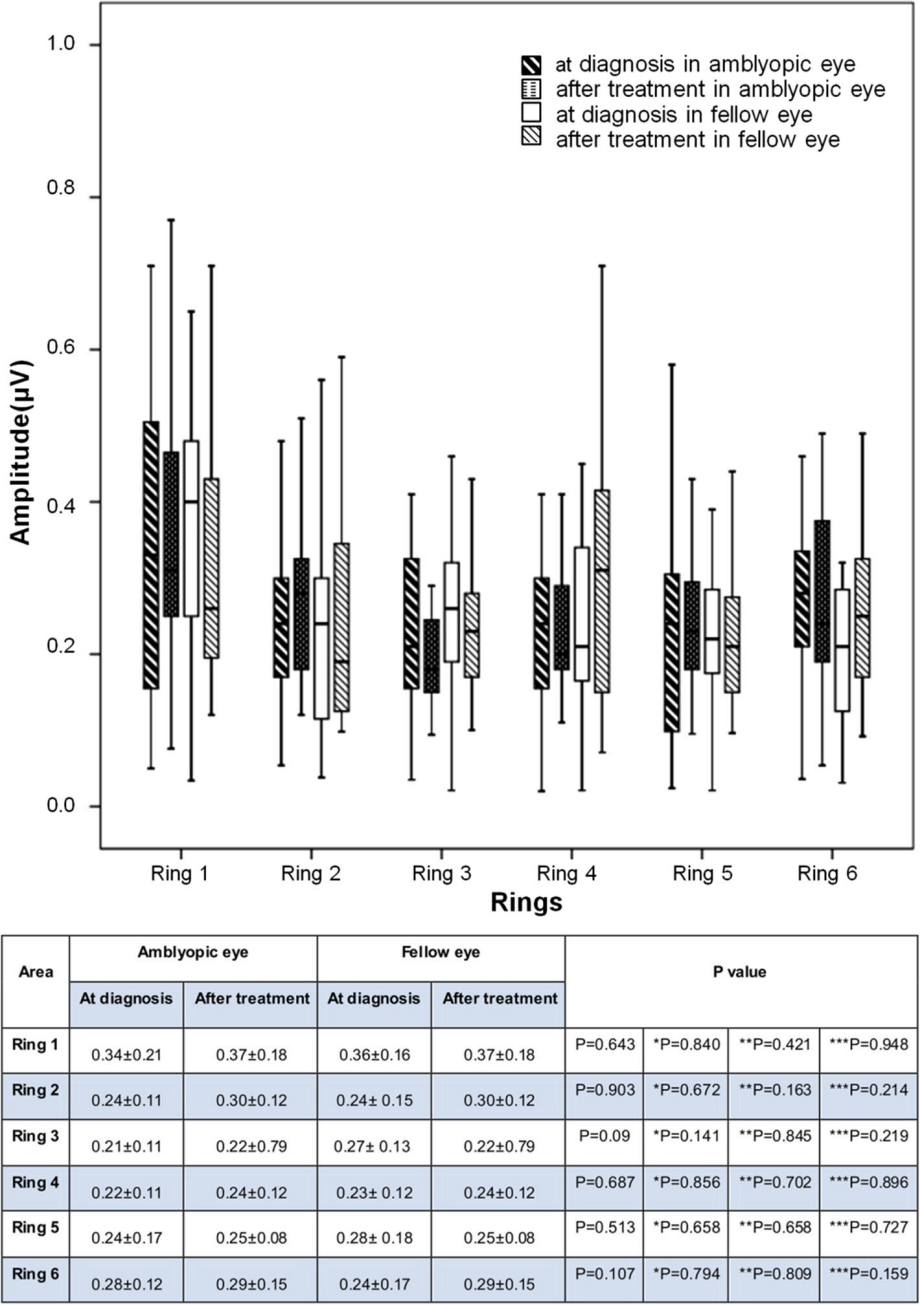
Comparison of multifocal visual evoked potential) (mfVEP) amplitudes for amblyopic eyes and fellow eyes in the six rings at the time of diagnosis and after occlusion treatment. There were no significant differences among the rings. Values are presented as mean ± SD (ms). *P* value derived from t-test for the comparison P: between amblyopic eyes and fellow eyes at the time of diagnosis *P:between amblyopic eyes and fellow eyes after treatment. **P: between at the time of diagnosis and after treatment in amblyopic eye ***P:between at the time of diagnosis and after treatment in fellow eye

The multifocal visual evoked potentials waveforms were classified and sorted according to the loci and eccentricities of their stimuli. In amblyopic eyes, the mean values of the each topographical rings did not differ significantly from each other at diagnosis (*p* = 0.391, Friedman test). After treatment, however, the value of ring 1 (central field) was significantly higher than those of the other rings (*p* = 0.001, Friedman test) (Fig. 2). The amplitudes in each rings at the time of diagnosis and after treatment in amblyopic eyes were shown in Fig. 3.

The comparison of multifocal visual evoked potentials amplitudes between amblyopic eyes and fellow eyes in the six rings at the time of diagnosis showed no significant difference (Wilcoxon sign rank test) (Fig. 3). Comparison of the amplitudes of the six rings between amblyopic eyes and fellow eyes after treatment also showed no significant difference (Wilcoxon sign rank test) (Fig. 3).

The difference in the amplitudes in each of the six rings at the time of diagnosis and after treatment in amblyopic eyes and fellow eyes did not differ significantly (Wilcoxon sign rank test) (Fig. 3).

**Fig. 4:**
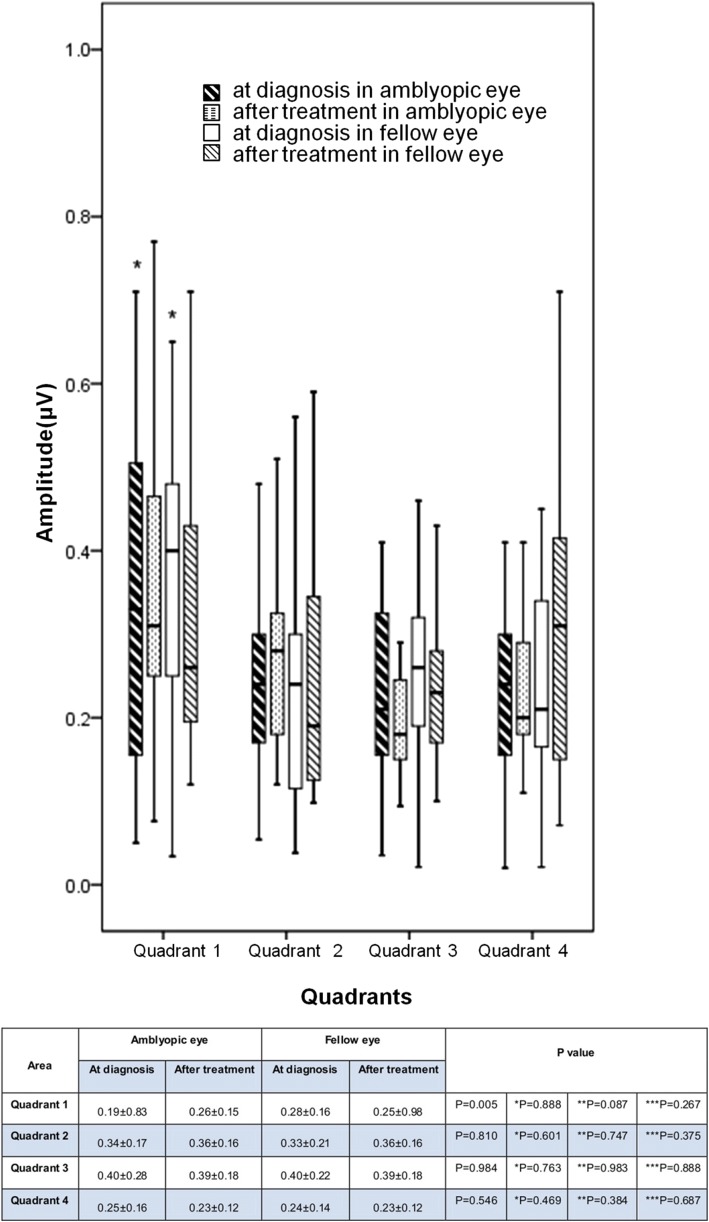
Comparison of multifocal visual evoked potentials (mfVEP) amplitudes for amblyopic eyes and fellow eyes in the four quadrants of the visual field at the time of diagnosis and after occlusion treatment. The values of quadrant 1 between amblyopic eyes and fellow eyes only show statistically significant differences at the time of diagnosis (*p* = 0.005) but not after treatment (*p* = 0.888). *quadrant 1 at diagnosis in amblyopic eyes and fellow eyes: statistically significant difference, *p* = 0.005. Values are presented as mean ± SD (ms). P value derived from t-test for the comparison P: between amblyopic eyes and fellow eyes at the time of diagnosis *P:between amblyopic eyes and fellow eyes after treatment. **P: between at the time of diagnosis and after treatment in amblyopic eye ***P:between at the time of diagnosis and after treatment in fellow eye.

The multifocal visual evoked potentials waveforms were also classified by four quadrants, divided by horizontal and vertical midlines, and then analyzed. The quadrant 1 values for the amblyopic versus fellow eyes were significantly different at the time of diagnosis (*p* = 0.005, Wilcoxon sign rank test) (Fig. 4), whereas after treatment there was no significant difference (*p* = 0.888, Wilcoxon sign rank test) (Fig. 4).

The difference in the amplitudes in the quadrants at diagnosis and after treatment in amblyopic eyes and fellow eyes also showed no significant difference (Wilcoxon sign rank test) (Fig. 4).

#### Latency

The comparison of latency values in each of the six rings at the time of diagnosis and after treatment in amblyopic and fellow eyes showed no significant differences (Table 2). The comparison of latencies for the six rings in amblyopic eyes and fellow eyes at diagnosis and after treatment showed no significant differences (Table 2).

**Table 2:** Comparison of latency values in each of the six rings in amblyopic and fellow eyes

Area	Amblyopic eye		Fellow eye		*P* value			
	At diagnosis	After treatment	At diagnosis	After treatment				
Ring 1	141.2 ± 20.8	147.3 ± 16.9	147.1 ± 17.5	136.2 ± 25.2	*P* = 0.221	*P = 0.155	**P = 0.46	***P = 0.12
Ring 2	144.0 ± 19.9	145.3 ± 13.3	142.9 ± 15.5	141.6 ± 19.0	*P* = 0.906	*P = 0.755	**P = 0.81	***P = 0.72
Ring 3	138.0 ± 20.5	144.3 ± 15.2	143.0 ± 17.1	140.8 ± 17.2	*P* = 0.334	*P = 0.384	**P = 0.56	***P = 0.98
Ring 4	141.7 ± 21.5	141.9 ± 16.3	137.6 ± 20.0	146.3 ± 17.5	*P* = 0.244	*P = 0.407	**P = 0.84	***P = 0.22
Ring 5	140.7 ± 16.6	145.6 ± 16.5	141.1 ± 19.3	139.5 ± 18.8	*P* = 0.950	*P = 0.187	**P = 0.31	***P = 0.55
Ring 6	144.1 ± 19.6	150.5 ± 13.0	135.4 ± 18.2	142.5 ± 19.9	*P* = 0.214	*P = 0.080	**P = 0.25	***P = 0.18
	^†^ *P* = 0.84	^†^ *P* = 0.48	^†^ *P* = 0.30	^†^ *P* = 0.65				

Values are presented as mean ± SD (ms)

*P* value derived from t-test for the comparison

*P*: between amblyopic eyes and fellow eyes at the time of diagnosis

*P:between amblyopic eyes and fellow eyes after treatment

**P: between at the time of diagnosis and after treatment in amblyopic eye

***P:between at the time of diagnosis and after treatment in fellow eye

^†^*P* value derived from t-test for the comparison between rings

The comparison of latencies for each of the four quadrants between the time of diagnosis and after treatment in amblyopic eyes and fellow eyes showed no significant differences (Table 3). The comparison of latencies for the four quadrants between amblyopic eyes and fellow eyes at diagnosis and after treatment also showed no significant differences (Table 3).

**Table 3:** Comparison of latencies for each of the four quadrants in amblyopic eyes and fellow eyes

Area	Amblyopic eye		Fellow eye		*P* value			
	At diagnosis	After treatment	At diagnosis	After treatment				
Quadrant 1	132.6 ± 21.3	141.4 ± 23.6	138.7 ± 22.4	141.8 ± 17.2	*P* = 0.35	*P = 0.95	**P = 0.22	***P = 0.63
Quadrant 2	141.5 ± 20.7	137.8 ± 24.2	135.3 ± 18.5	132.1 ± 24.1	*P* = 0.22	*P = 0.33	**P = 0.53	***P = 0.63
Quadrant 3	139.8 ± 19.4	142.2 ± 18.0	134.8 ± 20.0	140.1 ± 18.2	*P* = 0.40	*P = 0.68	**P = 0.68	***P = 0.41
Quadrant 4	142.9 ± 23.7	139.7 ± 23.8	134.9 ± 21.1	138.4 ± 22.1	*P* = 0.12	*P = 0.85	**P = 0.62	***P = 0.50
	^†^ *P* = 0.16	^†^ *P* = 0.66	^†^ *P* = 0.77	^†^ *P* = 0.20				

Values are presented as mean ± SD (ms)

*P* value derived from t-test for the comparison

*P*: between amblyopic eyes and fellow eyes at the time of diagnosis

*P:between amblyopic eyes and fellow eyes after treatment

**P: between at the time of diagnosis and after treatment in amblyopic eye

***P:between at the time of diagnosis and after treatment in fellow eye

^†^*P* value derived from t-test for the comparison between rings

## Discussion

Jeon et al. [16] reported that visual acuity quantification using absolute value of amplitude in pattern visual evoked potentials was useful in confirming subjective visual acuity. They found that the relationship between amplitude and logMAR acuity was linear. We also evaluated the correlation between visual acuity (logMAR) and amplitude or latency in multifocal visual evoked potentials. We found no significant relationship between visual acuity and amplitude or latency (linear regression: *P* = 0.271, 0.276, respectively). There were great variations of responses in mfVEP obtained from ring 1 in normal eye. Baseler et al. [8] suggested that the clinical utility of the mfVEP is limited because of the variation of responses obtained from identical locations in normal individuals. And Graham et al. and Hood et al. [17] suggested interocular comparison of mfVEP. Therefore we evaluated the effect of occlusion treatment of unilateral anisometropic amblyopia by correlating the differences in visual acuity before and after treatment in amblyopic eyes, and the difference between the two eyes to the equivalent difference in mfVEP parameters.

Kim et al. [18] reported that the multifocal visual evoked potentials were significantly greater in ring 1 than in the other five rings in normal adult controls. In a recent study, Jeon et al. [19] demonstrated, using multifocal visual evoked potentials, that the amplitudes of ring 1 of the anisometropic amblyopic eyes were not significantly different from those of the other rings before treatment. After occlusion treatment, however, the amplitude of ring 1 in the amblyopic eyes exhibited a significantly greater changes than the other rings, suggesting that increased VA in amblyopic eyes are associated with improved visual function in the central field. In our study, the amplitudes in ring 1 of the amblyopic eyes were significantly greater after treatment than those of the other rings. This finding is consistent with the results of Yu et al. [20] who demonstrated that visual acuity as more severely impaired in the foveal area than in the periphery of amblyopic eyes. A possible explanation for this phenomenon is that the center of the visual field, which vision is the clearest region and it demands an accurately focused image for development, whereas the periphery of the visual field is less clear region and it requires a less accurately focused image.

The most prominent deficit in amblyopia is in spatial vision, as measured by either Snellen acuity or grating acuity. The amblyopes also show decreased contrast sensitivity and visual discrimination ability. The same ranges of characteristics were revealed by experimentally created amblyopia in the macaque monkeys [21]. It has been suggested that anisometropic and strabismic amblyopia do not originate from a common pathophysiological process. The high spatial frequency (low temporal frequency) losses are inferred to represent parvocellular pathway deficits and lower spatial frequency (higher temporal frequency) losses are inferred to represent magnocellular pathway deficits. They have hypothesized that the distinction between the two types of amblyopia depends on the severity of magnocellular or parvocellular visual pathway defects [22]. We also think anisometropic and strabismic amblyopia might have similar neural anomalies even though they have different etiologies (chronic unilateral blur vs chronic unilateral suppression). The results would depend on the severity of magnocellular or parvocellular visual pathway defects than types of amblyopia.

Shan et al. [23] suggested that anisometropic amblyopia is primarily associated with an abnormal parvocellular visual system, rather than an abnormal magnocellular visual system. Parvocellular pathways tend to reflect the visual function of the fovea and account for the relatively greater defects observed in central visual function than is seen with peripheral visual function in amblyopic individuals [24]. Therefore, in this study, a significant change of amplitude in the central field in the amblyopic eye after treatment might reflect improvement of the abnormal parvocellular visual system function.

Another study showed a significant response latency difference between the amblyopic and normal eyes which the responses in the central region of the visual field (rings 1 and 2) had a longer latency in amblyopic eyes than normal eyes [25]. Most of the patients showed severe degree of anisometopia (6/60–6/9). In our study, however, the comparison of the latencies in each of the six rings and each of the four quadrants showed no significant difference between the amblyopic eyes and fellow eyes. This discrepancy may be due to the mild degree of anisometropia seen in our cases (20/100 in one patient, Table 1) or lack of optimization in the process not using multiple stimuli in our study.

A number of studies have examined the associations among the degree of anisometropia, baseline visual acuity, and final visual acuity in patients with anisometropic amblyopia [25]. Kutschke et al. [26] reported that the degree of anisometropia is not related to the baseline visual acuity but, rather, to the final visual acuity of amblyopic eye. In this study, we compared amplitude and latency values of each of six rings and four quadrants of amblyopic eyes versus fellow eyes after treatment. In quadrant 1, the amplitudes were significantly lower in amblyopic eyes than in fellow eyes at the time of diagnosis. After treatment, however, this parameter was no longer significantly different. These statistical changes in quadrant 1 may reflect the improvement in visual acuity (i.e., final visual acuity).

We evaluated the effect of occlusion treatment on amblyopia using multifocal visual evoked potentials to compare anisometropic amblyopic eyes and fellow eyes. There was no significant difference between the amplitudes for each ring in anisometropic amblyopic eyes and fellow eyes at the time of diagnosis and after treatment. The values for quadrant 1 in amblyopic eyes were significantly lower than those of fellow eyes at the time of diagnosis but showed no significant differences after treatment, suggesting that in quadrant 1 the amblyopic eyes improved from the pre-treatment baseline (*p* = 0.087). The occlusion treatment and the plasticity caused quadrant 1 in the amblyopic eyes to become similar to those of their fellow eyes. The fibers from the macula occupy the temporal portion. This papillomacular bundle is highly sensitive to visual function, and quadrant 1 is included in the superotemporal area [27]. There have been a report that the amplitudes in the multifocal visual evoked potentials (RETIscan® system, Roland, Brandenburg, Germany) were larger in the inferior field than superior field. [18] The subtle difference in quadrant 2 (inferotemporal) may not be reflected to the relatively large amplitude in the inferior field. Therefore, in our study, the quadrant 2 did not show treatment effect like quadrant 1, even though it is also included in the papillomacular bundle.

It has been reported that the change in amplitude on the central field (ring 1) in amblyopic eyes could be a useful, objective monitoring method for observing the improvement in VA [16]. However, the multifocal visual evoked potentials results might prove to have low reproducibility among tests and individuals [28]. Large-amplitude noise can be analyzed as the neural response if the electrical responses are too small. Therefore, it could be a better method for monitoring the effect of amblyopic treatment with quadrant 1 because it (superotemporal region) is a larger area than that of ring 1. Comparisons using the values of quadrant 1, rather than those of ring 1, in patients whose visual acuity was improved after treatment will help quantify the amplitude changes even when considering the fixation unreliability of multifocal visual evoked potentials. Our study also confirmed the larger changes in the central responses than the peripheral responses with less severely impaired eye.

There were some limitations to our study. First, because of the relatively small number of patients, we could not analyze amplitude changes based on the degree of anisometropia. Second, because the test might have a learning curve and patient compliance could differ according to age, additional studies of correlations among age, follow-up period, and total numbers of tests are needed. The interpretation of results must also take the effects of unstable fixation during measurements into consideration. The fixation stability is crucial for good VEP results, and it may be more useful in cooperative children. The usefulness of mfVEP in pre-verbal children should be evaluated by further study. Third, we did not take into consideration on the effect with stimulus size, and we use only the 16 checks/segments. Recording responses to more than one check size, to make sure the optimal response in the amblyopia is not obtained [13, 28]. Therefore, no significant difference is found for any of the latency comparisons. (Tables 2,3) It must be considered with caution in this study.

The standard method of visual acuity assessment in cooperative children is a standard letter acuity tests, and these tests are subjective tests. Clinicians have difficulty confirming objective visual acuity whether it is in the course of visual and cognitive development. We also need objective method to evaluate the level of underlying organic dysfunction in patients with nonorganic overlay superimposed upon real dysfunction. Therefore mfVEP can be used as an objective VA test in cooperative children with amblyopia treatment and malingering even though it takes a longer time. In the present study, we focused on multifocal visual evoked potentials amplitude, which could be considered a useful, objective measurement to replace visual acuity testing. Comparing the results of the multifocal visual evoked potentials recordings of amblyopic eyes with those of fellow eyes can be an effective method for verifying an abnormality.

## Conclusion

In conclusion, the multifocal visual evoked potentials have the advantage that it produces a topographical measure of damage compared with standard visual evoked potentials. Using multifocal visual evoked potentials, we found changes of smaller dysfunctional areas in anisometropic amblyopia after treatment using hundreds of stimulations presented in the same amount of time. Changes in the differences between amblyopic eyes and fellow eyes in quadrant 1 could be a useful, objective method for monitoring improvements in visual acuity even when taking fixation reliability into consideration.

## Additional file

Additional file 1: Raw data: amplitude and latency in mfVEP. (XLS 54 kb)

## Abbreviations

BCVA: Best corrected visual acuity
D: Diopter
mfVEP: (multifocal visual evoked potential)
VA: Visual acuity
